# Hospital-Wide Sepsis Detection: A Machine Learning Model Based on Prospectively Expert-Validated Cohort

**DOI:** 10.3390/jcm15020855

**Published:** 2026-01-21

**Authors:** Marcio Borges-Sa, Andres Giglio, Maria Aranda, Antonia Socias, Alberto del Castillo, Cristina Pruenza, Gonzalo Hernández, Sofía Cerdá, Lorenzo Socias, Victor Estrada, Roberto de la Rica, Elisa Martin, Ignacio Martin-Loeches

**Affiliations:** 1 Multidisciplinary Sepsis Unit, Intensive Care Unit, Son Llatzer University Hospital, 07198 Palma de Mallorca, Spain; agiglioj@gmail.com (A.G.); marandacorreo@gmail.com (M.A.); asocias@hsll.es (A.S.); adelcastillo@hsll.es (A.d.C.); lsocias3@gmail.com (L.S.); 2 Multidisciplinary Sepsis Group, Health Research Institute of the Balearic Islands (IdISBa), 07198 Palma de Mallorca, Spain; roberto.delarica@gmail.com; 3 Balearic Islands University (UIB), 07198 Palma de Mallorca, Spain; 4 Faculty of Medicine, Finis Terrae University, 7501015 Santiago, Chile; 5 Critical Care Department, Clinica Las Condes Hospital, 7591046 Santiago, Chile; 6 Instituto de Ingenieria del Conocimiento (IIC), 28049 Madrid, Spain; cristina.pruenza@iic.uam.es (C.P.); gonzalo.hernandez.1293@gmail.com (G.H.); sofycm93@gmail.com (S.C.); elisa.martin@iic.uam.es (E.M.); 7 Informatic Service, Son Llatzer University Hospital, 07198 Palma de Mallorca, Spain; vestrada@hsll.es; 8 Department of Intensive Care Medicine, Multidisciplinary Intensive Care Research Organization (MICRO), St. James’s Hospital, D08 NHY1 Dublin, Ireland; drmartinloeches@gmail.com

**Keywords:** sepsis, machine learning, natural language processing, artificial intelligence, clinical decision support systems, random forest, big data, hospital medicine

## Abstract

**Background/Objectives:** Sepsis detection remains challenging due to clinical heterogeneity and limitations of traditional scoring systems. This study developed and validated a hospital-wide machine learning model for sepsis detection using retrospectively developed data from prospectively expert-validated cases, aiming to improve diagnostic accuracy beyond conventional approaches. **Methods:** This retrospective cohort study analysed 218,715 hospital episodes (2014–2018) at a tertiary care centre. Sepsis cases (n = 11,864, 5.42%) were prospectively validated in real-time by a Multidisciplinary Sepsis Unit using modified Sepsis-2 criteria with organ dysfunction. The model integrated structured data (26.95%) and unstructured clinical notes (73.04%) extracted via natural language processing from 2829 variables, selecting 230 relevant predictors. Thirty models including random forests, support vector machines, neural networks, and gradient boosting were developed and evaluated. The dataset was randomly split (5/7 training, 2/7 testing) with preserved patient-level independence. **Results:** The BiAlert Sepsis model (random forest + Sepsis-2 ensemble) achieved an AUC-ROC of 0.95, sensitivity of 0.93, and specificity of 0.84, significantly outperforming traditional approaches. Compared to the best rule-based method (Sepsis-2 + qSOFA, AUC-ROC 0.90), BiAlert reduced false positives by 39.6% (13.10% vs. 21.70%, *p* < 0.01). Novel predictors included eosinopenia and hypoalbuminemia, while traditional variables (MAP, GCS, platelets) showed minimal univariate association. The model received European Medicines Agency approval as a medical device in June 2024. **Conclusions:** This hospital-wide machine learning model, trained on prospectively expert-validated cases and integrating extensive NLP-derived features, demonstrates superior sepsis detection performance compared to conventional scoring systems. External validation and prospective clinical impact studies are needed before widespread implementation.

## 1. Introduction

Sepsis remains one of the most significant challenges in hospital care due to its high morbidity, mortality, and considerable clinical heterogeneity [1]. Characterised by a dysregulated host response to infection, sepsis can rapidly progress to severe organ dysfunction and septic shock [2]. Traditional detection strategies—such as the Systemic Inflammatory Response Syndrome (SIRS) criteria, quick Sequential Organ Failure Assessment (qSOFA), and Sequential Organ Failure Assessment (SOFA) criteria—demonstrate limited sensitivity and specificity, frequently leading to diagnostic delays and suboptimal clinical outcomes [3]. These methods heavily depend on clinician judgement and often miss early or atypical presentations.

Recent expert perspectives emphasise the need for advanced artificial intelligence (AI) and machine learning (ML) tools that integrate clinical, biochemical, and microbiological data to improve sepsis detection and outcomes globally [4]. However, current artificial intelligence and machine learning (AI-ML) models face significant limitations. Many rely heavily on retrospective sepsis identification through discharge coding, resulting in high false-negative rates and reduced real-time clinical applicability [5]. Furthermore, most studies utilise predominantly two databases—MIMIC-II/III/IV and the eICU Collaborative Research Database, which lack prospective validation of sepsis cases at clearly defined points in time [6,7]. Additionally, these models are usually constrained to specific hospital settings, such as emergency departments or ICUs, neglecting broader, system-wide complexities. Importantly, current AI-ML models largely overlook valuable clinical insights from unstructured data sources like physician notes. In this area, natural language processing (NLP) has considerable potential for improvement [8].

This study addresses these gaps by developing and validating a ML model for sepsis detection using retrospective data augmented with prospectively validated sepsis diagnoses, identified in real-time by a dedicated expert unit. This initiative emerges from multidisciplinary collaboration among clinicians, intensivists, data scientists, biomedical engineers, informatics specialists, and academic researchers. The primary goal is to enhance sepsis detection accuracy throughout hospital environments by leveraging expert-confirmed sepsis cases beyond the limitations of discharge coding. The secondary objective explores the added value of incorporating unstructured clinical data through NLP to improve model performance and real-world applicability.

## 2. Materials and Methods

### 2.1. Study Design and Setting

This study was designed as a non-concurrent cohort study utilising retrospective data from a hospital-wide Electronic Health Record (EHR) system, spanning five years from 1 January 2014 to 31 December 2018. The dataset included all patients aged 14 years and older admitted to any clinical area within the hospital, including the emergency department (ED), general medical and surgical wards, and the intensive care unit (ICU). This approach allowed a comprehensive evaluation of sepsis identification models across the full spectrum of hospital care settings, reflecting the heterogeneity of real-world clinical practice.

Patients aged 14 years and older were included, which corresponds to the population managed as adults, both for inclusion in Sepsis Protocol strategies as well as by the Multidisciplinary Sepsis Unit at our institution. Paediatric patients (age < 14 years) were excluded from this study as they are managed through separate clinical protocols and were not subject to prospective validation by the adult-focused MSU during the study period. No other exclusion criteria were defined.

The study was conducted at Hospital Universitario Son Llàtzer (HUSLL), a 450-bed tertiary care facility in Spain. HUSLL serves as a referral centre for approximately 250,000 people and is equipped with an 18-bed multidisciplinary ICU and advanced laboratory and informatics infrastructure. The hospital has maintained a long-standing commitment to sepsis care through its established Multidisciplinary Sepsis Unit (MSU) and has implemented a structured, hospital-wide Electronic Sepsis Protocol since 2006. The hospital developed its own Automated Sepsis Detection System (ADS) in 2010, providing a rich, high-resolution clinical data environment for sepsis research.

A distinguishing feature of this study is the integration of real-time, prospective case validation by the MSU. Between 2014 and 2018, all potential sepsis cases were identified and validated in real-time by expert clinicians as they occurred. This prospective clinical adjudication process ensured that the ground truth labels used for model training were clinically verified at the time of diagnosis, rather than relying solely on retrospective administrative coding. The ML model itself was subsequently developed retrospectively using these prospectively validated cases, following a retrospective model development approach as described by Futoma et al. [9] and Bedoya et al. [10].

### 2.2. Ethical Considerations

The Ethics and Health Research Committee of the Balearic Community (CEIC-Ib) ID 463721 approved the study in June 2021, which was conducted in accordance with European and Spanish data protection regulations, as well as in compliance with the Declaration of Helsinki. Given the retrospective nature of the model development and the use of anonymised patient records, the requirement for individual patient consent was waived. The hospital and patients retain ownership of the dataset under current legislation, making it inaccessible for external distribution. Furthermore, the BiAlert model holds a patent and has received approval from the European Medicines Agency (6 June 2024) as a medical device, preventing it from being open-source.

### 2.3. Endpoints

The study’s primary endpoint was the accurate identification of sepsis cases using a ML-based predictive model, validated against real-time, prospectively confirmed diagnoses by a multidisciplinary expert unit. The model’s performance was primarily evaluated using the area under the receiver operating characteristic curve (AUC-ROC).

Secondary endpoints included:

- Comparison between ML models and traditional rule-based systems
- Performance across various clinical settings (ED, ICU, wards)
- Impact of natural language processing (NLP)-based integration of unstructured data on diagnostic accuracy
- Reduction in false-positive rates compared to conventional approaches

### 2.4. Sepsis Case Identification and Validation

Sepsis case identification was conducted through a real-time prospective validation process led by the Multidisciplinary Sepsis Unit (MSU) throughout the entire study period (2014–2018). The process followed a structured two-step methodology.

Initially, potential cases were flagged through one or more of six detection pathways: (1) an automated rule-based alert system incorporating SIRS criteria and 11 additional laboratory abnormalities; (2) clinical suspicion documented in the electronic health record (EHR); (3) positive microbiological cultures; (4) physiological deterioration triggering automated alerts; (5) direct physician consultation; and (6) systematic screening of all ICU admissions.

Patients identified through any of these pathways underwent real-time expert validation by the MSU within hours of detection. This validation was based on a modified Sepsis-2 criterion that required evidence of systemic inflammatory response (SIRS criteria) plus organ dysfunction consistent with severe sepsis or septic shock, as defined by the 2012 Surviving Sepsis Campaign guidelines [11]. MSU activation criteria and protocol can be reviewed in Borges-Sa et al. article [12]. The approach combines the sensitivity of Sepsis-2 with emphasis on organ dysfunction to ensure diagnostic rigour, thereby reducing false negatives while maintaining clinical validity.

The timestamp of MSU validation served as the reference point for sepsis diagnosis. For model training purposes, only clinical data from the 48 h preceding this timestamp were used, ensuring that the model could not access information occurring after the diagnosis was made. This temporal restriction is critical to prevent data leakage and ensure the model’s predictions would be clinically actionable in real-time implementation.

Although Sepsis-3 criteria were available from 2016 onward, the decision to use modified Sepsis-2 was based on: (1) consistency across the entire 2014–2018 study period, (2) the established MSU workflow already in place since 2006, and (3) preliminary analysis showing that Sepsis-3 (SOFA-based) criteria yielded lower sensitivity in our population. Sepsis-3 criteria were nonetheless evaluated as a separate rule-based model for comparison purposes (see Results).

### 2.5. Data Sources and Feature Extraction

#### 2.5.1. Electronic Health Record Integration

Data used for model development were extracted from an integrated hospital-wide EHR system encompassing both structured and unstructured data sources. Structured data included: laboratory results (HP-Doctor system), vital signs (HP-Doctor system), demographic variables (Gacela platform), pharmacy records, and triage assessments. Unstructured data sources comprised free-text clinical documentation, including physician notes, nursing notes, emergency department reports, and triage narratives, written in Spanish and Catalan.

Additionally, data from two hospital-specific systems were integrated: (1) the Electronic Sepsis Protocol (EPS), in use since 2006, which captures structured clinical variables based on Surviving Sepsis Campaign criteria; and (2) the Automated Sepsis Detection System (ADS), implemented in 2010, which generates a sepsis likelihood score using 15 predefined clinical and biochemical variables.

#### 2.5.2. Feature Extraction and Engineering

A total of 2829 variables were extracted from the EHR system. Of these, 2038 (72.03%) were structured variables derived directly from laboratory systems, vital sign monitors, and clinical databases. The remaining 791 (27.96%) were unstructured variables extracted from free-text clinical documentation using natural language processing (NLP) techniques.

For structured data, dynamic trends of key physiological and biochemical markers were engineered by calculating rates of change over time. For each patient, current values were compared with historical baselines from the preceding six months when available. The rate of change (slope) was calculated using at least one prior measurement. For patients without historical data (e.g., first hospital encounter), the first recorded value was used as the baseline, and no delta was calculated, effectively assuming a normal prior state.

For unstructured text data, NLP was performed using wrapper techniques and the Dunning log-likelihood test to identify clinically relevant terms from Spanish and Catalan free-text notes [7]. Extracted terms were categorised according to clinical signs and symptoms, anatomical locations, and comorbidities based on ICD-10 classification. This automatic mapping was validated by clinical experts to ensure accuracy and relevance. The complete list of NLP-derived variable categories is provided in Supplementary Materials.

The NLP-derived variables and structured clinical data were internally standardised using a manifest-catalogue system based on SNOMED CT, LOINC, and ATC medical terminology standards to ensure semantic consistency and facilitate potential interoperability with other healthcare information systems.

#### 2.5.3. Temporal Windowing

For patients with confirmed sepsis (SE group), all available data from the 48 h period immediately preceding the MSU validation timestamp were extracted. This ensured that the model would learn from clinically relevant information available before the diagnosis was confirmed, mimicking real-world early detection scenarios.

For non-septic patients (NSE group), data from the first 48 h of their hospital episode were extracted. This approach provided a comparable temporal window while avoiding selection bias that might occur if a random 48 h period were chosen (e.g., selecting periods near discharge when patients are clinically stable).

### 2.6. Variable Selection and Preprocessing

#### 2.6.1. Statistical Variable Selection

As a preliminary step, univariate statistical analysis was performed to identify variables potentially associated with sepsis. The Mann–Whitney-Wilcoxon (MWW) test, a non-parametric test suitable for non-normally distributed data, was applied to assess whether each variable’s distribution differed significantly between the WSE and NSP groups. A significance threshold of *p* < 0.01 was used. *p*-values were subsequently adjusted using the Holm correction to control the family-wise error rate (raw and adjusted *p*-values are provided in Supplementary File. Variables with *p* ≥ 0.01 were not automatically excluded, as ML algorithms might uncover multivariate interactions not apparent in univariate analysis.

Multicollinearity among candidate variables was assessed, and highly correlated redundant variables were flagged for review by the clinical team. However, the final variable selection was primarily driven by ML algorithms’ intrinsic feature selection capabilities rather than manual exclusion based on correlation alone.

#### 2.6.2. Machine Learning-Based Feature Selection

In addition to statistical pre-selection, wrapper-based feature selection techniques were employed. These methods allowed ML algorithms to automatically select the most predictive variables during the training process. Random forest algorithms, for example, inherently rank variables by importance based on their contribution to predictive accuracy across decision trees. Support vector machines and neural networks also implicitly weight features during training.

Through this iterative process, the ensemble of ML models converged on a set of 230 variables deemed most relevant for sepsis prediction. Of these, 62 (26.95%) were structured variables (61 continuous numerical variables plus sex as a binary categorical variable), and 168 (73.04%) were derived from unstructured NLP text data. This distribution highlights the substantial contribution of free-text clinical documentation to the predictive model.

#### 2.6.3. Data Preprocessing

To prepare data for ML algorithms, several preprocessing steps were undertaken:

1. Missing Data Handling: The primary algorithms used in this study (random forests and gradient boosting models) can handle missing values natively through surrogate splits and by treating missingness as informative. For algorithms that require complete data (such as support vector machines and certain neural network implementations), missing values were imputed using reference normal values derived from the distribution of non-septic patients. The percentage of episodes with available measurements for each variable is reported in Supplementary Table S1.
2. Outlier Management: Extreme outliers were identified and reviewed by the clinical team. Physiologically implausible values due to measurement or recording errors were corrected or excluded. Legitimate extreme values (e.g., very high lactate in septic shock) were retained.
3. Normalisation: For the 61 continuous structured variables, Z-score normalisation was applied to standardise the scale across different measurement units. The mean and standard deviation were calculated from the training set only and then applied to both training and test sets to prevent data leakage. Z-score transformation centres the mean at zero, with values above the original mean becoming positive and values below becoming negative. This normalisation ensures that variables with different units (e.g., heart rate vs. creatinine) contribute equally to distance-based algorithms such as support vector machines.
4. Text Preprocessing: For NLP variables, standard text processing steps included tokenisation, removal of stopwords, and extraction of clinically meaningful terms using the Dunning test. Categorical variables derived from text (e.g., presence of specific symptoms or anatomical sites) were binary encoded.

### 2.7. Machine Learning Model Development

#### 2.7.1. Software and Implementation

ML model development was conducted by the Instituto de Ingeniería del Conocimiento (IIC, Madrid, Spain) using Python-based libraries (version 3.8). Statistical analyses were performed using R (version 3.6). Due to intellectual property considerations related to the EMA-approved medical device status of the BiAlert Sepsis model, specific library versions and proprietary algorithmic implementations are not disclosed but are available upon reasonable request to IIC for academic collaboration purposes.

#### 2.7.2. Algorithms Evaluated

Multiple supervised ML algorithms were evaluated for their ability to predict sepsis from the available features:

1. Random Forest (RF): An ensemble of decision trees that reduces overfitting through bootstrap aggregation and random feature selection at each split. Random forests are robust to missing data and can capture complex non-linear interactions.
2. Support Vector Machines (SVM): A classification algorithm that finds the optimal hyperplane separating classes in a high-dimensional feature space. SVMs are effective in high-dimensional settings and can model non-linear relationships through kernel functions.
3. Neural Networks (NN): Multi-layer perceptrons with non-linear activation functions capable of learning complex hierarchical representations of data.
4. Gradient Boosting (GB): An ensemble technique that sequentially builds decision trees, with each tree correcting errors made by previous trees. Gradient boosting often achieves high predictive accuracy but requires careful tuning to avoid overfitting.

Each algorithm was trained independently using the training dataset, and hyperparameters were optimised through cross-validation (see below).

#### 2.7.3. Ensemble Learning Strategy

To improve robustness and predictive performance, ensemble learning techniques were employed. Individual ML models were combined using a majority voting strategy. For a given patient episode, each model in the ensemble generated a binary prediction (sepsis or no sepsis). The final ensemble prediction was determined by simple majority vote: the class receiving the most votes was selected as the ensemble’s output.

The best-performing standalone ML ensemble was designated as ML-IIC (Machine Learning—Instituto de Ingeniería del Conocimiento), which served as the reference ML model for subsequent comparisons.

### 2.8. Model Categories and Ensemble Strategy

A total of 30 distinct models were developed and evaluated, categorised into four groups:

#### 2.8.1. Category 1: Rule-Based Clinical Scoring Systems

Traditional fixed-rule models based on established sepsis criteria were implemented as comparators:

1. Sepsis-2: SIRS criteria (≥2 of: temperature > 38 °C or <36 °C, heart rate > 90 bpm, respiratory rate > 20/min, WBC > 12,000 or <4000/μL) plus suspected or confirmed infection [13].
2. Sepsis-3 (SOFA): Sequential Organ Failure Assessment score ≥ 2 points in the presence of infection, according to the Third International Consensus Definitions for Sepsis and Septic Shock [1].
3. qSOFA: Quick SOFA score ≥ 2 points (systolic BP ≤ 100 mmHg, respiratory rate ≥ 22/min, altered mentation).
4. Sepsis-2 + qSOFA: Combined criteria requiring both Sepsis-2 and qSOFA positivity.
5. Local ADS Model: The hospital’s pre-existing Automated Sepsis Detection System using 15 fixed clinical and biochemical variables.

#### 2.8.2. Category 2: Pure Machine Learning Models

Multiple algorithms were trained independently and in various ensemble combinations using the 230 selected variables:

- Individual Random Forest models with varying hyperparameters
- Individual Support Vector Machine models
- Individual Neural Network architectures
- Individual Gradient Boosting models
- Ensemble combinations of the above using majority voting

The ML-IIC model emerged as the best-performing pure machine learning ensemble from this category.

#### 2.8.3. Category 3: Hybrid Models (ML + Rule-Based Ensembles)

Models were combined with traditional rule-based systems to create hybrid predictive models. In these ensembles, the ML-IIC model’s output was combined with the output of rule-based scoring systems through majority voting:

- BiAlert Sepsis (formerly BISEPRO): ML-IIC + Sepsis-2 criteria
- ML-IIC + Sepsis-3 (SOFA)
- ML-IIC + qSOFA
- ML-IIC + Sepsis-2 + qSOFA
- Additional combinations with local ADS scores

#### 2.8.4. Category 4: Alternative Ensemble Configurations

Additional ensemble configurations were tested, including weighted voting schemes and alternative combinations of rule-based systems. A total of 30 models across all categories were systematically evaluated.

### 2.9. Model Training and Validation

#### 2.9.1. Dataset Partitioning

The complete dataset of 218,715 patient episodes was randomly divided into two subsets while preserving patient-level independence. Specifically, patients with multiple hospital episodes during the study period were assigned entirely to either the training or test set, ensuring that no patient contributed data to both sets. This approach prevents data leakage and provides a more realistic estimate of model performance on new, unseen patients.

The dataset was partitioned as follows:

- Training Set: 5/7 of episodes (n = 145,539 episodes, approximately 71.4%)
- Test Set: 2/7 of episodes (n = 58,216 episodes, approximately 28.6% remaining, with final totals accounting for 14,960 excluded episodes)

The prevalence of sepsis was similar in both sets (approximately 5.4%), and stratification by outcome was not necessary due to the large sample size.

#### 2.9.2. Cross-Validation During Training

Within the training set, 4-fold cross-validation was employed to optimise hyperparameters and assess model stability. The training set was divided into 4 subsets (folds). In each iteration, 3 folds were used for training, and the remaining fold was used for validation. This process was repeated 4 times, with each fold serving as the validation set once. The arithmetic mean of performance metrics across all 4 iterations provided an estimate of model performance and guided hyperparameter tuning.

Cross-validation was applied only to the training set (5/7 of the data). The test set (2/7 of the data) was held out entirely and used only once for final model evaluation, ensuring an unbiased estimate of generalisability.

#### 2.9.3. Model Selection and Final Evaluation

For each of the 30 models, performance was evaluated on the held-out test set. Predictions were generated for all 58,216 test episodes, and performance metrics were calculated. 

Because the test set is large (>58,000 episodes including >3000 sepsis cases), a single evaluation provides a stable and reliable estimate of model performance. Random seed values were fixed during model training to ensure reproducibility of results within the development environment.

### 2.10. Statistical Analysis and Performance Metrics

#### 2.10.1. Performance Metrics

Model performance was assessed using standard binary classification metrics calculated from confusion matrices: area under the receiver operating characteristic curve (AUC-ROC), sensitivity, specificity, positive predictive value (PPV), negative predictive value (NPV), and false positive rate.

#### 2.10.2. Statistical Comparisons

For univariate feature selection, the Mann–Whitney-Wilcoxon test was used to compare variable distributions between WSE and NSP groups, with a significance threshold of *p* < 0.01. Comparisons between model performance metrics were assessed using appropriate statistical tests for paired proportions. Odds ratios (OR) with 99% confidence intervals were calculated for key clinical variables and model performance comparisons. Given the large test set size (>58,000 episodes), even small differences in performance metrics achieved statistical significance (*p* < 0.01).

#### 2.10.3. Reproducibility

All statistical and ML analyses were conducted using reproducible computational pipelines. R scripts for statistical analysis and Python code for machine model development were version-controlled. Due to the proprietary nature of the EMA-approved BiAlert Sepsis model, full code cannot be publicly shared; however, methodological details are provided to enable independent replication of the approach.

#### 2.10.4. Model Calibration

Model calibration was assessed using the Brier score, which measures the mean squared difference between predicted probabilities and observed outcomes. The Brier score quantifies the accuracy of probabilistic predictions, with values closer to 0 indicating better calibration.

### 2.11. Artificial Intelligence Use Statement

During the preparation of this work, the authors used Claude.AI to improve the redaction of this paper. After using this tool, the authors reviewed and edited the content as needed and takes full responsibility for the content of the publication.

## 3. Results

### 3.1. Patient Cohort and Episode Distribution

**Figure 1:**
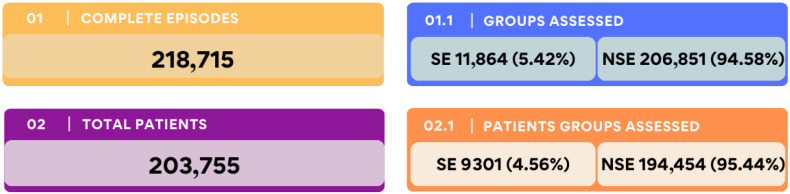
Population Distribution in the Study Cohort. Distribution of hospital episodes and patients classified as with sepsis episodes (SE) or non-septic patients (NSE) during the study period.

A total of 203,755 patients contributed to 218,715 patient episodes during the study period. Among these, MSU prospectively confirmed 9301 patients (4.56%) and 11,864 episodes (5.42%) as meeting the criteria for sepsis or septic shock (SE). These episodes were classified as with sepsis episodes (SE) or non-septic patients (NSE), as shown in Figure 1 (specific detection strategy is available in Supplementary Figure S1).

More than half of the episodes (54.1%) were recorded in the emergency department (ED), with the remaining 45.9% occurring in general wards and the ICU. However, the proportion of sepsis cases was significantly lower in the ED than in other hospital settings, with an incidence of 2.87% versus 14.84%, respectively (OR: 0.1698 [0.1627 to 0.1773], *p* < 0.001) (Table S3 in the Supplementary Materials).

SE patients were older than those without sepsis. The mean age in the SE group was 68.48 years (99% CI: 68.07–68.89), compared to 47.82 years (99% CI: 47.71–47.94) in the NSE group (OR: 1.005 [1.002–1.009]; *p* < 0.01). Male patients were significantly more prevalent than female patients across the entire cohort (*p* < 0.01), with no significant difference in the male-to-female ratio between the septic and non-septic groups (OR 0.999 [0.959–1.042, *p* = 0.996]). Table 1 presents overall data. (Supplementary Table S1 in the Supplementary Materials provides additional demographic and clinical features).

### 3.2. Data Sources and Variable Selection

**Figure 2:**
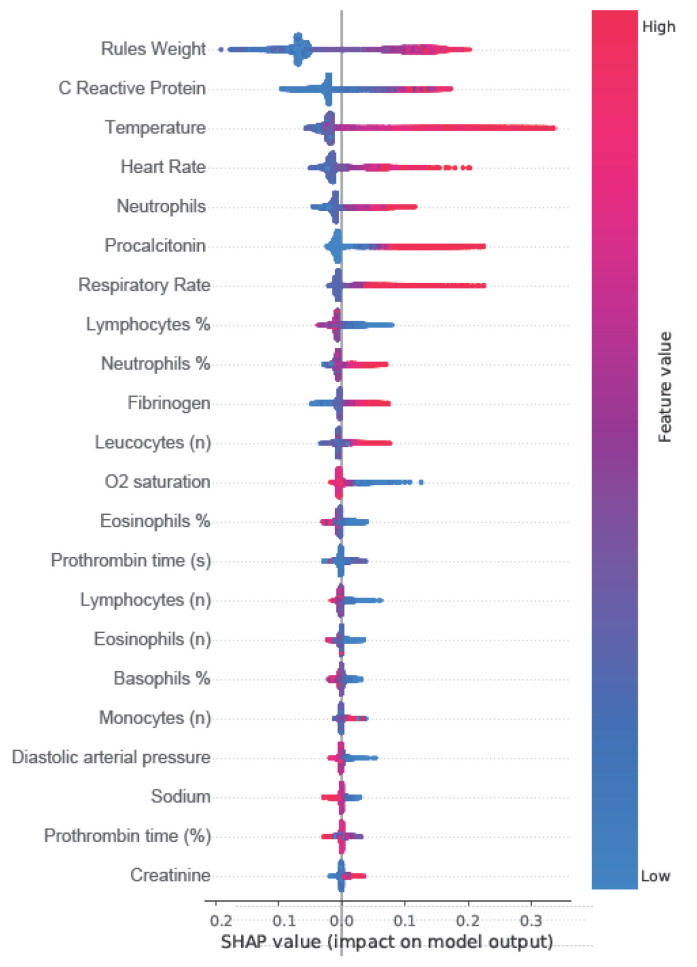
SHAP Value Plot for Key Predictive Variables in the BiAlert Sepsis Model. SHAP (Shapley Additive Explanations) plot showing the top 21 relevant structured variables and rule model weights in the BiAlert Sepsis ensemble model. Features are listed in descending order of importance based on mean absolute SHAP values across all patients. Each data point represents a feature’s contribution to the model output for an individual prediction, coloured by a standardised measurement value: red indicates elevated measurements, while blue indicates lower measurements. Important features include Rules Weight, C Reactive Protein, Temperature, and Heart Rate, with high values of these parameters contributing positively to sepsis probability.

From the electronic health record (EHR) system, 2829 variables were extracted for analysis. Of these, 2038 variables (72.03%) were structured, and 791 (27.96%) were unstructured (*p* < 0.01). Among all extracted variables, 230 (8.13%) were selected as relevant for sepsis identification. Most of the selected variables (168, 73.04%) were derived from unstructured data, while 62 (26.95%) were structured (OR: 13.5021 [10.0106 to 18.2113], *p* < 0.001), as shown in Supplementary Figure S2. Figure 2 shows the Shapley additive explanations (SHAP) plot with the 21 most relevant structured variables and rules model weights for the ensembled models.

As detailed in Table 2, the primary sources of structured data were laboratory results (46.87%), triage assessments (26.65%), and pharmacy records (22.72%). The most frequently captured structured clinical variables were heart rate, systolic and diastolic blood pressure, and body temperature. Leukocyte count was the most commonly recorded laboratory parameter.

Several biomarkers were significantly associated with sepsis episodes, including eosinopenia (OR: 1.323 [1.211–1.447], *p* < 0.01) and hypocalcaemia (OR: 1.155 [1.048–1.272], *p* < 0.01). In contrast, commonly used variables in traditional scoring systems, such as mean arterial pressure (MAP) (OR: 0.979 [0.956–1.003], *p* = 0.08), Glasgow Coma Scale (GCS) (OR: 0.994 [0.810–1.220], *p* = 0.95), and platelet count (OR: 1.002 [1.0–1.004], *p* = 0.04) were not significantly associated with SE (Supplementary Table S2). Notably, many variables identified as predictive of SE are not currently included in standard fixed-rule sepsis diagnostic tools or widely used critical care scoring systems.

### 3.3. Predictive Model Performance

Thirty predictive models were developed and assessed. Across all comparisons, the ML models outperformed the traditional fixed-rule approaches. Among the conventional methods, the combination of Sepsis-2 and qSOFA yielded the highest performance, with an area under the receiver operating characteristic curve (AUC-ROC) of 0.90.

**Figure 3:**
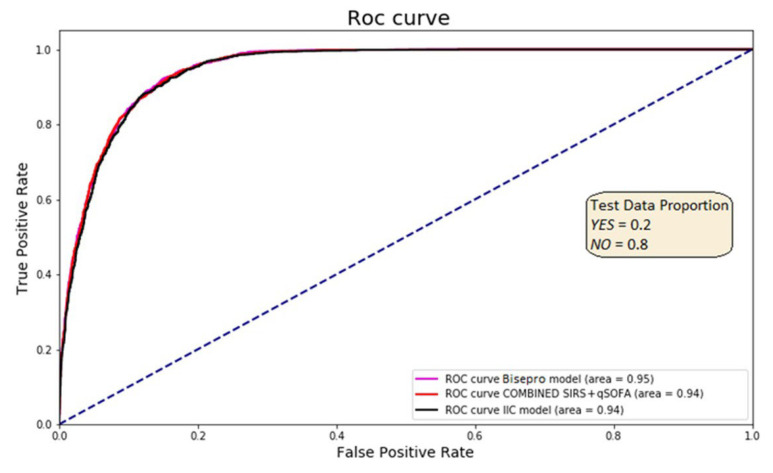
ROC curve from the Test analysis group for ML predictive methods. The pink curve corresponds to the best combined model which was BiAlert Sepsis (formerly BISEPRO), the red curve corresponds to the ML-IIC model combined with SEPSIS-2 + qSOFA, and the black curve corresponds to the standalone model without combination with the reference, ML-IIC.

The highest-performing ML model was based on a random forest algorithm (ML-RF), which achieved a sensitivity of 0.93 and specificity of 0.83. When this model was integrated with the Sepsis-2 criteria to form the BiAlert Sepsis model, the specificity improved to 0.84 while maintaining the same sensitivity (OR: 69.75 [64.94–74.92], *p* < 0.01). Incorporating qSOFA into this ensemble (BiAlert Sepsis + qSOFA) did not lead to additional performance gains, with a sensitivity and specificity of 0.93 and 0.83, respectively. The BiAlert Sepsis model demonstrated good calibration with a Brier score of 0.0699. The AUC-ROC was 0.95 for BiAlert Sepsis and 0.94 for both ML-RF alone and BiAlert Sepsis + qSOFA, with no statistically significant differences between the three ML-based models (Figure 3).

The BiAlert Sepsis model produced a lower false-positive rate (13.10%) than the best fixed-rule strategy (Sepsis-2 + qSOFA: 21.70%), representing a relative reduction of 39.6% in false alarms (OR: 0.552 [0.505–0.604], *p* < 0.01). The complete performance results for all models are summarised in Table 3.

## 4. Discussion

This study presents a hospital-wide, ML-based model for detection, developed retrospectively using prospectively expert-validated cases as ground truth. The model demonstrated superior diagnostic performance compared to traditional fixed-rule approaches, achieving an AUC-ROC of 0.95 while reducing false-positive rates by nearly 40% compared to the best-performing conventional system. Importantly, sepsis was more frequently identified in older adults and outside the emergency department, particularly in general wards and ICU, highlighting the need for hospital-wide surveillance solutions. These findings reflect real-world sepsis epidemiology and demonstrate that ML can enhance diagnostic precision and improve detection across diverse care settings.

A central strength of this study is its reliance on real-time prospective sepsis validation using a modified Sepsis-2 definition that includes organ dysfunction to better align with Sepsis-3 specificity while retaining Sepsis-2 sensitivity [1]. Unlike previous models that rely on discharge coding, this approach minimises misclassification and anchors the diagnostic timestamp to clinically verified events [14,15]. Sepsis detection was triggered through six independent clinical systems, further improving case capture and reducing detection bias [16,17]. The broad inclusion of episodes across emergency, ICU, and general ward settings enhances the model’s applicability, distinguishing it from earlier studies restricted to single departments. This aligns well with recent insights highlighted by Martin-Loeches et al., emphasising the complexities and challenges of timely sepsis diagnosis and management across diverse clinical presentations [4].

It is important to distinguish between prospective case validation and prospective model validation. In our study, sepsis cases were identified and validated by expert clinicians in real-time (2014–2018), providing high-quality ground truth labels. The ML model was subsequently developed retrospectively using these validated cases.

The integration of ML enabled the utilisation of 230 predictive variables, substantially surpassing the median of 22 typically employed in conventional rule-based systems [17]. Remarkably, several variables identified by the ML model as highly relevant, such as eosinopenia, hypoalbuminaemia, and hypocholesterolaemia, are absent from standard sepsis scoring systems such as Sepsis-2 and Sepsis-3. Conversely, traditional variables, including mean arterial pressure (MAP), Glasgow Coma Scale (GCS), and platelet count, showed minimal association with sepsis in our cohort, despite their established roles in traditional and many AI-driven models [1,18]. These findings suggest that ML not only uncovers novel clinical insights previously overlooked but also fundamentally challenges the established paradigms in sepsis diagnostics.

A notable innovation of our model lies in the integration of structured and unstructured data through natural language processing (NLP), with over 70% of the 230 variables derived from unstructured clinical documentation. Prior studies by Yang et al. (2023) and Horng, S et al. (2017) reported significant improvements in predictive accuracy (AUC-ROC from 0.67 to 0.86) by incorporating NLP-derived features [19,20]. Our results reinforce and extend these findings, highlighting NLP’s critical role of NLP in capturing clinically valuable information that structured data alone might miss.

Several hospital-wide sepsis ML models exist, including TREWS, Epic Sepsis Model, Sepsis Watch, and COMPOSER [21,22]. Our contribution lies in using prospectively expert-validated cases rather than discharge coding, and extensively integrating NLP-derived features (73% of variables) to achieve high performance (AUC-ROC 0.95) with 40% fewer false positives than traditional systems.

This study has several limitations. First, single-centre design may limit generalisability; external validation is needed. Second, data from 2014 to 2018 may not reflect current practice, particularly post-COVID-19 changes. Third, NLP trained on Spanish/Catalan limits international transferability. Fourth, subgroup analyses by demographics and clinical settings were not performed and should be addressed. Fifth, while using prospectively validated cases provides superior ground truth compared to discharge coding, this remains a retrospective model development study; prospective validation assessing clinical impact on patient outcomes is required before widespread implementation. Finally, missing data and the gradual nature of sepsis may introduce subtle biases despite robust algorithms.

An important feature is the regulatory approval of the BiAlert Sepsis model. As of June 2024, the European Medicines Agency (EMA) approved the model as a medical device and granted an official patent. Although this status prevents the model from being open-source, it marks a significant advancement by bridging the gap between algorithm development and real-world clinical implementation. Regulatory certification ensures that the model adheres to rigorous safety and efficacy standards, supporting its potential adoption into clinical decision support systems and hospital infrastructure. The recognition of ML algorithms as medical products is essential for their acceptance, accountability, and integration into the health system.

## 5. Conclusions

In summary, this retrospective study introduces a hospital-wide machine learning model trained on prospectively expert-validated cases that significantly outperforms conventional systems in terms of accuracy and specificity for sepsis detection. Through prospective clinical validation, integration of unstructured data, and broad applicability across hospital settings, the BiAlert Sepsis Model represents a meaningful step forward in precision diagnostics for sepsis. The model’s recognition of the model as a certified medical device reinforces its clinical relevance and readiness for integration into practice. Future work should focus on multicentre validation and continuous monitoring to adapt the model across different healthcare environments and further improve the outcomes of sepsis care.

## Figures and Tables

**Table 1:** Quantitative structured variables from septic and non-septic patients.

Variables	Sepsis Patients					Non-Septic Patients				
	Mean	Median	St. Desv	Episodes (%)	IC 99%	Mean	Median	St. Desv	Episodes (%)	IC 99%
Clinical Variables										
Age (years)	68.48	72	17.19	11,864 (100%)	68.07–68.89	47.82	44	20.27	206,851 (100%)	47.71–47.94
Heart Rate	98.75	101	23.29	11,075 (93.35%)	98.18–99.32	82.85	80	17.83	182,306 (88.13%)	82.74–82.96
Respiratory Rate	20.68	20	6.07	7336 (61.83%)	20.49–20.86	16.91	16	3.36	101,852 (49.24%)	16.89–16.94
GCS (value)	14.92	15	0.75	1708 (14.4%)	14.87–14.97	14.97	15	0.44	18,443 (8.92%)	14.96–14.97
FiO_2_ (%)	36.32	28	21.14	3034 (25.57%)	35.33–37.31	26.37	21	15.11	14,857 (7.18%)	26.05–26.69
MAP (mmHg)	58.62	59.33	5.24	3233 (27.25%)	58.38–58.86	60.17	61.67	4.62	7955 (3.85%)	60.04–60.31
Temperature (°C)	37.15	36.9	1.23	10,989 (92.62%)	37.1–37.18	36.3	36.2	0.64	165,872 (80.19%)	36.3–36.31
Biochemical Variables										
Metabolic										
Albumin (g/dL)	2.99	3	0.63	2290 (19.3%)	2.95–3.02	3.56	3.56	0.6	9049 (4.37%)	3.55–3.58
Bicarbonate (mEq/L)	24.67	24.4	6.47	1269 (10.7%)	24.2–25.14	25.75	25.9	5.91	2363 (1.14%)	25.44–26.06
Cholesterol (mg/dL)	128.64	122	53.01	1190 (10.03%)	124.6–132.6	171.05	166	52.73	7772 (3.76%)	169.5–172.59
Blood glucose (mg/dL)	150.78	127	83.79	10,537 (88.81%)	148.6–152.89	113.91	101	48.45	98,372 (47.56%)	113.5–114.3
LDH (U/L)	318.35	227	687.57	3498 (29,48%)	288.4–348.3	214.16	189	166.37	17,293 (8.36%)	210.9–217.42
Total Proteins (g/dL)	5.71	5.7	0.99	1800 (15.17%)	5.65–5.77	6.4	6.4	0.78	8649 (4.18%)	6.38–6.42
Biomarkers										
Lactate (mmol/L)	2.32	1.7	1.94	1077 (9.08%)	2.16–2.47	1.77	1.29	1.53	771 (0.37%)	1.63–1.91
Procalcitonin (ng/mL)	12.22	1.37	120.85	4737 (39.93%)	7.7–16.75	0.72	0.1	3.22	1646 (0.8%)	0.52–0.92
C Reactive Protein (mg/L)	157.53	147.6	100.68	10,095 (85.09%)	154.95–160.1	32.37	7.5	56.52	54,988 (26.58%)	31.74–32.99
Renal										
Creatinine (mg/dL)	1.43	1.11	1.11	10,886 (91.76%)	1.41–1.46	0.93	0.81	0.56	99,953 (48.32%)	0.92–0.93
Sodium (mEq/L)	137.47	137.7	5.55	10,811 (91.12%)	137.3–137.6	139.32	139.6	3.36	99,393 (48.05%)	139.3–139.35
Urea (mg/dL)	61.83	49	43.19	10,837 (91.34%)	60.76–62.9	36.72	32	22.69	99,137 (47.93%)	36.53–36.9
Hepatic Profile										
Direct Bilirubin (mg/dL)	2.03	1.04	2.68	1975 (16.65%)	1.87–2.18	1.34	0.66	2.27	4989 (2.41%)	1.26–1.43
Haematological Variables										
Blood Count										
Leukocytes (×10^9^/L)	14.38	13.6	9.33	10,927 (92.1%)	14.15–14.61	9.74	9.04	4.22	105,469 (50.99%)	9.71–9.77
Neutrophils (×10^9^/L)	11.66	10.9	7.17	10,848 (91.44%)	11.49–11.84	6.59	5.79	3.57	105,394 (50.95%)	6.56–6.62
Lymphocytes (×10^9^/L)	1.41	1.05	4.91	10,848 (91.44%)	1.29–1.53	2.1	1.96	1.85	105,395 (50.95%)	2.09–2.12
Eosinophils (%)	0.82	0.32	1.59	10,856 (91.5%)	0.78–0.86	1.83	1.3	1.99	105,398 (50.95%)	1.82–1.85
Platelets (×10^9^/L)	238.26	218	127.43	10,865 (91.58%)	235.1–241.41	244.67	236	80.2	105,420 (50.96%)	244.04–245.3
Haemoglobin (g/dL)	11.89	12	2.28	10,857 (91.51%)	11.83–11.94	13.43	13.6	1.93	105,419 (50.96%)	13.42–13.45
Haematocrit (%)	36.68	36.9	7.02	10,855 (91.5%)	36.51–36.85	40.56	41	5.72	105,419 (50.96%)	40.52–40.61
Coagulation										
PT act (%)	65.99	68	20.72	9473 (79.85%)	65.44–66.54	87.12	90	19.78	76,107 (36.79%)	86.94–87.3
aPTT (Seg)	33	31.5	8.16	8350 (70.38%)	32.77–33.23	31.86	31.1	5.94	68,729 (33.23%)	31.8–31.92
Fibrinogen (mg/dL)	685.14	674	216.3	8053 (67.88%)	678.9–691.35	472.99	444	150.85	64,737 (31.3%)	471.47–474.52

**Table 2:** Variable sources and association with septic (SE) patients.

Origin/Sources	Number	% of Total	Relevant Variables	% Variables Associated SE
Sepsis Code	15	0.50%	15	100%
Vital signs	54	1.91%	6	11.11%
Analytical variables	1326	46.87%	45	3.39%
Triage	754	26.65%	112	14.85%
Pharmacy data	643	22.72%	14	2.17%
ED Reports	37	1.30%	37	100%
TOTAL	2829	100%	229	8.09%

**Table 3:** Model performances for sepsis detection. First three models represent the three best performance fixed-rules models and the three best performing machine learning models represent one of only ML ensembles and two ML plus fixed-rules ensembles.

	Sepsis-2	Sepsis-3 (SOFA)	Sepsis-2 + qSOFA	ML-IIC	ML-IIC + Sepsis-2 + qSOFA	ML-ICC + Sepsis-2 (BiAlert Sepsis)
AUC	0.89	0.85	0.90	0.94	0.94	0.95
SENSIB	0.88	0.81	0.93	0.93	0.93	0.93
SPECIF	0.77	0.80	0.73	0.83	0.83	0.84
TP (%)	17.60	17.10	18.60	18.70	18.60	18.70
TN (%)	61.60	59.20	58.20	66.20	66.40	66.80
FP (%)	18.40	20.80	21.70	13.70	13.60	13.10
FN (%)	2.40	2.90	1.50	1.40	1.40	1.40

## Data Availability

Data is unavailable due to privacy or ethical restrictions.

## Supplementary Materials

The following supporting information can be downloaded at: https://www.mdpi.com/article/10.3390/jcm15020855/s1, Extended Methodology section; List S1: Classification according signs and symptoms as category general by CIE-10; List S2: Classification according to anatomical parts and tissues by CIE-10; Table S1: Complete dataset from septic and non septic patients; Table S2: Structured variables associated with episodes of SE/SS (WSE group); Table S3: Relationship between included patients and the presence or absence of SE/SS according to whether they presented in the ED or the rest of the hospital; Table S4: *p*-values of the variables; Figure S1: Origin of patients according to alert systems and electronic medical records; Figure S2: Types of variables included: structured or unstructured (NLP).

## Abbreviations

The following abbreviations are used in this manuscript:

AI	Artificial Intelligence
ADS	Automated Sepsis Detection System
aPTT	Activated Partial Thromboplastin Time
AUC-ROC	Area Under the Receiver Operating Characteristic Curve
BP	Blood Pressure
CEIC-Ib	Ethics and Health Research Committee of the Balearic Community
ED	Emergency Department
EHR	Electronic Health Record
EMA	European Medicines Agency
EPS	Electronic Sepsis Protocol
FN	False Negative
FP	False Positive
GB	Gradient Boosting
GCS	Glasgow Coma Scale
HUSLL	Hospital Universitario Son Llàtzer
ICD-10	International Classification of Diseases, 10th Revision
ICU	Intensive Care Unit
IIC	Instituto de Ingeniería del Conocimiento
LDH	Lactate Dehydrogenase
MAP	Mean Arterial Pressure
ML	Machine Learning
ML-IIC	Machine Learning—Instituto de Ingeniería del Conocimiento
ML-RF	Machine Learning—Random Forest
MSU	Multidisciplinary Sepsis Unit
NLP	Natural Language Processing
NPV	Negative Predictive Value
NSE	Non-Septic Episodes
OR	Odds Ratio
PPV	Positive Predictive Value
PT	Prothrombin Time
qSOFA	Quick Sequential Organ Failure Assessment
RF	Random Forest
SE	Sepsis Episodes
SHAP	Shapley Additive Explanations
SIRS	Systemic Inflammatory Response Syndrome
SINOMED CT	Systematised Nomenclature of Medicine Clinical Terms
SOFA	Sequential Organ Failure Assessment
SVM	Support Vector Machines
TN	True Negative
TP	True Positive
WBC	White Blood Cell
