# Supplementary material for: Hospital-Wide Sepsis Detection: A Machine Learning Model Based on Prospectively Expert-Validated Cohort
Source: J Clin Med. 2026 Jan 21;15(2):855. doi: 10.3390/jcm15020855 (PMC12841928; doi:10.3390/jcm15020855)
Supplement: Supplementary file 1 [file jcm-15-00855-s001.zip › jcm-4041736-supplementary.pdf]

Hospital-Wide Sepsis Detection: A Machine Learning Model Based on Prospectively Expert-Validated Cohort.

Authors:

Marcio Borges-Sa [1,2,3,*,] Andres Giglio [1,2,3,4], Maria Aranda [1,2], Antonia Socias [1,2], Alberto del Castillo [1,2], Cristina Pruenza [5], Gonzalo Hernández [5], Sofía Cerdá [5], Lorenzo Socias [1,2,3], Victor Estrada [6], Roberto de la Rica [2], Elisa Martin [5] and Ignacio Martin-Loeches [7]

Affiliations:

1 Multidisciplinary Sepsis Unit, Intensive Care Unit, Son Llatzer University Hospital, Palma de Mallorca, Spain

2 Multidisciplinary Sepsis Group of the Health Research Institute of the Balearic Islands, IdISBa

3 Balearic Islands University (UIB), Palma de Mallorca, Spain

4 Critical Care Department, Finis Terrae University and Clínica Las Condes Hospital Faculty of Medicine, Santiago, Chile

5 Instituto de Ingenieria del Conocimiento (IIC), Madrid, Spain

6 Informatic Service, Son Llatzer University Hospital, Palma de Mallorca, Spain

7 Department of Intensive Care Medicine, Multidisciplinary Intensive Care Research Organization (MICRO), St. James's Hospital, Dublin, Ireland

Supplementary Data:

I. Extended Methodology section

Setting

This study was conducted at Son Llàtzer University Hospital in Palma de Mallorca, Spain, which has 450 beds, of which 18 are ICU beds. We included patients over 14 years old from the ED, from all medical and surgical hospitalization areas, and the ICU.

Study Population

For the inclusion of patients with sepsis, we have utilized the definition known as SEPSIS-2, with severe sepsis (SE) and septic shock (SS), as it was the one used in the hospital during the study period (4,18).

All detected episodes of SE and SS have been included, labeled, and prospectively validated using a form developed at the hospital that has been in use since 2006. There is an Integrated Electronic SE/SS Care Protocol (EPS), where each physician can voluntarily include patients over 14 years old that reflect the SE/SS criteria. Over the years, an intrahospital culture has been created and strengthened with educational updates, posters, and regular sessions for all doctors and nurses, both general and by service, for the early identification and attention of patients with SE/SS, called Sepsis Code (18,23).

Additionally, those patients identified in the Automated SE/SS Detection Program (ADS) and subsequently validated as positive for SE/SS were included in the patient cohort.

Study Design

With the objective of improving the detection of patients with SE/SS as well as minimizing false positive (FP) and false negative (FN) cases, an observational study with retrospective analysis was conducted, but with prospective validation of cases, applying predictive techniques from BD-AI-ML. To address this need, different models were implemented and combined, resulting in an ensemble approach. In this way, patients diagnosed with SE/SS were detected and included in databases that prospectively obtained information from the Electronic Health Record (EHR) (®HP-Doctor). With the goal of decreasing FP cases and thus reducing the number of false alerts, all patients diagnosed with SE/SS by the hospital's various doctors were subsequently reviewed by the Multidisciplinary Sepsis Unit (MSU) team to confirm or discard the diagnoses. This team used a combination of clinical, microbiological, radiological, and case evolution criteria (18).

The study was carried out by the clinical and informatics research group of Son Llàtzer University Hospital (which constitutes the Sepsis Group of the Fundació Institut d'Investigació Sanitària Illes Balears - IDISBA) together with the group of experts from the Institute of Knowledge Engineering (IIC) of the Autonomous University of Madrid. Clinical researchers were responsible for designing the study, reviewing cases, considering the relevance of the included variables, and excluding variables not related to the septic process. The IIC group collaborated in the study design, developed the ensemble predictive model based on BD-AI-ML methodology, descriptive analysis, and the statistical validation of the

approaches. Finally, both groups analyzed all variables, the results, and wrote the present article.

Authorization was obtained from the hospital management to conduct the study with its corresponding European and Spanish Data Protection Law, after review by the Legal Departments of the Hospital and IDISBA, as well as by the Hospital's Research Commission. The study was approved by the Ethics and Health Research Committee of the Balearic Community (CEIC-Ib), as well as the authorization was obtained.

Data collection

In this study, various data sources available in the EH) were utilized, where all variables have been stored prospectively and combined to achieve the best prediction approximations based on the application of BD-AI-ML techniques. The data sources used come from different repositories: analytical variables (®HP-Doctor), clinical variables (Gacela program on ®HP-Doctor), EPS, ADS, ED and Triage reports. The first two databases are therefore obtained from the EHR of the ®HP-Doctor commercial brand.

The EPS allows any hospital doctor to include a patient over 14 years old suspected of SE/SS based on the criteria defined in the Surviving Sepsis Campaign (SSC) of 2012 (4). In it, patients must have at least two SIRS criteria and one or more of organic dysfunction. In the procedure, once a patient was included, the MSU assessed and began clinical follow-up of the same in any area of the hospital from detection until resolution, death, or discharge of the patient (18).

The ADS Program is a hospital's own developed computerized alert system for possible SE/SS (18). This system uses 15 clinical and analytical variables, from which a score is obtained that sums the weighted value of each variable (from 0 to 4). If a patient has a score of 6 or more points, they are included in a list that the MSU team can access to perform the timely evaluation in real-time. This system tracks all patients over 14 years old in all hospital areas (including ED and ICU) and can be generated immediately each time it is triggered by an MSU physician.

With this procedure, the data set was divided into two groups for analysis: 1) With SE/SS (WSE), and 2) Without such diagnosis (NSP). To this end, we used all the available information of the patients in the hospital, including the hospital area where they have been identified. The goal was to better classify the different populations, for which a division is made according to the services where the patient was at the time of SE/SS case detection.

1. The WSE group is formed by those patients who were included in the EPS or those classified as such in the ADS, as they are exclusive programs to each other. All these cases, that is, WSE patients, were reviewed and followed prospectively by the MSU.
2. The NSP group includes those patients classified as such in the ADS along with patients without alert during the study period. Thus, patients considered WITHOUT sepsis are patients with an alert labeled as No Sepsis and the rest of the patients WITHOUT an alert for possible sepsis. There was no additional criterion.

The study focuses, therefore, on the analysis of the WSE and NSP groups of patients with or without sepsis, respectively, admitted to all hospital areas, excluding pediatric patients (under 14 years old), in the period from January 2014 to December 2018.

Statistical Analysis

As preliminary work, variables potentially relevant for distinguishing sepsis cases from non-sepsis cases were statistically identified. Initially, a statistical test was applied, specifically the Mann–Whitney–Wilcoxon (MWW) test. Subsequently, a set of clinical and analytical variables was analyzed using a significance level of 0.01. Although this step was not intended as a formal variable selection procedure, adjustment for multiple testing was performed using the Holm correction to control the family-wise error rate (29,30).

Quantitative calibration assessment was made using the Brier score, which measures the mean squared difference between predicted probabilities and observed outcomes and directly reflects the accuracy of probabilistic risk estimation. In our model, the Brier score was 0.0699, indicating good overall agreement between predicted probabilities and observed outcomes.

Furthermore, other so-called wrapper techniques have been used, allowing prediction approximations to automatically select variables from the different sources used. Lastly, given the characteristics of the variables in repositories containing free texts written in Spanish such as reports and triage, the application of basic Natural Language Processing (NLP) techniques such as the Dunning test was required to obtain relevant information from the texts (30,31).

In the SE/SS prediction phase, two AI disciplines are considered: 1) a knowledge-based system, which will be considered as the reference model, in which exclusively the variables used in the EPS with the known criteria (1,4,18) were utilized; and 2) AI predictive models capable of reproducing domain knowledge from observations. These latter models have the disadvantage of not retaining the acquired knowledge in an explicit form, such as rules, and are generally less interpretable for an end-user who wants to understand the model's outputs.

In the initial phase used in both disciplines, a preprocessing of the data was carried out. The data were cleaned with the objective of obtaining quality data to include in the predictive models. To avoid scaling problems, a Z-Score normalization was performed, which measures how far a data point is from the mean of the distribution. For this, the mean of the distribution is centered on zero, and those values that were above the mean in the original database with normalization will have positive values, and those that were below will take negative values.

The total sample was divided into two datasets. A 5/7 proportion of the total records chosen at random would constitute the training set and the rest of the records, 2/7 would form the test set.

Different ML algorithms have been used to predict SE/SS using AI predictive models.: Neural Networks, Support Vector Machines, Gradient Boosting, Random Forest, among others. The core concept of employing different techniques is to average the results obtained by various noisy or weak models in a weighted manner with majority voting to develop a

robust model, termed an ensemble. Averaging reduces the variance existing among the different weak models. Prediction for a new case/patient is carried out by presenting the new case to all models and the label that receives the majority of occurrences is reported as the prediction (majority vote).

For the validation of the models' accuracy during the training phase, the k-fold cross-validation technique was applied. Here, the data from the training set (5/7 of the total) were divided into k subsets. In turns, one of the subsets is used as test data and the rest (k-1) as training data. The cross-validation process is repeated for k iterations, with each of the subsets used as test data in turn. Finally, the arithmetic mean of the results from each iteration is calculated to obtain a global result that allows verification of the model's accuracy in each case.

Complementary Documents:

List S1
Classification according signs and symptoms as category general by CIE-10 divided in 20 categories

01 - Cardiovascular: ICD-10 (R00-R09) Murmur, arrhythmia, extrasystole, angina
02 - Ear, Nose, Throat, and Mouth: ICD-10 (R00-R09) Cough, sneeze, expectoration, sputum
03 - Gastrointestinal: ICD-10 (R10-R19) Burning sensation, vomiting, diarrhea, peritonitis
04 - Integumentary: ICD-10 (R20-R23) Abrasion, ulceration, urticaria, eczema
05 - Neurological: ICD-10 (R40-R46) Stupor, disorientation, confusion
06 - Obstetric/Gynecological: ICD-10 (R30-R39) Vaginitis, amenorrhea, infertility, vaginism
07 - Ocular: ICD-10 (R40-R46) Vision changes, flashes, spot
08 - Psychiatric: ICD-10 (R40-R46) Aggression, suicidal, depressive, anxiety
09 - Pulmonary: ICD-10 (R00-R09) Dyspnea, choking, emphysema, wheezing
10 - Rheumatologic: ICD-10 (R25-R29) Lower back pain, synovitis, contracture, dorsal pain
11 - Urological: ICD-10 (R30-R39) Hematuria, dysuria, urination, cystitis
12 - Sensory: Specific (captures patient sensations that do not apply in other categories) (In ICD-10 corresponds to R50-R69) Prickling, chills, tremors, burning sensation
13 - Fever: Specific (a key symptom for differentiating febrile from afebrile patients, thus it warrants its own category) (In ICD-10 corresponds to R50-R69) Fever, febrile, low-grade fever
14 - Pain: Specific (a key symptom for differentiating patients presenting with pain, thus it warrants its own category) (In ICD-10 corresponds to R50-R69) Pain, painful
15 - Hemorrhage: Specific (considered useful as a differential symptom and is not captured in other categories) (In ICD-10 corresponds to R50-R69) Hemorrhage, metrorrhagia, bleeding, epistaxis
16 - Infection: Specific (a key symptom for differentiating patients presenting with an infection, and is not specifically captured in other categories) (In ICD-10 corresponds to R50-R69) Infection, infected, infectious
17 - Hormonal: Specific (there are hormonal symptoms that are not captured in other categories) (In ICD-10 corresponds to R83-R89) Thyroiditis, hirsutism, hypothyroidism, hyperthyroidism
18 - Inflammation: Specific (considered useful as a differential symptom and is not captured in other categories) (In ICD-10 corresponds to R83-R89) Inflammation, swelling, megalia, tumefaction
19 - Suppuration: Specific (considered useful as a differential symptom and is not captured in other categories) (In ICD-10 corresponds to R83-R89) Suppurating, exudate, purulent
20 - Blood Components: Specific (considered useful as a differential symptom and is not captured in other categories) (In ICD-10 corresponds to R70-R79) Anemia, iron deficiency

List S2:
Classification according to anatomical parts and tissues by CIE-10 divided in 17 categories, any examples of each group

1. Cardiovascular System: heart, arteries, arterioles, venules, blood vessels
2. Digestive System: esophagus, pancreas, liver
3. Excretory System: urethra, colon, bladder
4. Reproductive System: genital, testicle, prostate, fallopian tube
5. Respiratory System: larynx, trachea, lungs
6. Articular System: joint, wrist, ankle, elbow
7. Endocrine System: thyroid, gland, pituitary
8. Skeletal System: femur, ulna, metacarpal
9. Immune System: lymphocytes, antibodies
10. Integumentary System: skin, nails, hair, epidermis
11. Muscular System: contractility, neuromuscular, triceps
12. Nervous System: synapse, neuronal, neurotransmitter
13. Lymphatic System: lymph, lymphatic
14. Head: eyebrow, head, jaw
15. Trunk: abdomen, thorax, flank
16. Upper Extremities: forearm, hand
17. Lower Extremities: foot, leg

In total, there were 20 possible categories for symptoms and 17 for anatomy.

SUPLEMENTARY TABLES AND FIGURES

Table S1. Complete dataset from septic and non septic patients:

Clinical and analytical structured variables after data preprocessing. The first column indicates the variable, the next five columns show the mean values, median, standard deviation, number of complete episodes, and the 99% confidence interval for 11,864 (5.42%) episodes related to SE/SS (WSE group) and 206,851 (94.58%) to No Sepsis (NSE group).

Variables	Septic Patients (WSE)					Non-septic patients (NSE)				
	Mean	Median	St. Desv	Episodes (%)	IC 99%	Mean	Median	St. Desv	Episodes (%)	IC 99%
Clinical Variables										
Age (years)	68,48	72	17,19	11864 (100,0%)	68,07-68,89	47,82	44	20,27	206851 (100,0%)	47,71 - 47,94
Heart Rate*	98,75	101	23,29	11075 (93,35%)	98,18-99,32	82,85	80	17,83	182306 (88,13%)	82,74 - 82,96
Respiratory Rate *	20,68	20	6,07	7336 (61,83%)	20,49-20,86	16,91	16	3,36	101852 (49,24%)	16,89 - 16,94
GCS (value)	14,92	15	0,75	1708 (14,4%)	14,87 - 14,97	14,97	15	0,44	18443 (8,92%)	14,96 - 14,97
FiO2 (%)	36,32	28	21,14	3034 (25,57%)	35,33 - 37,31	26,37	21	15,11	14857 (7,18%)	26,05 - 26,69
SatVMO2 (%)	93,52	96	6,51	10893 (91,82%)	93,36 - 93,68	97,49	98	3,32	151446 (73,22%)	97,47 - 97,52
MAP (mmHg)	58,62	59,33	5,24	3233 (27,25%)	58,38 - 58,86	60,17	61,67	4,62	7955 (3,85%)	60,04 - 60,31
DAP (mmHg)	65,47	65	14,83	11060 (93,22%)	65,11 - 65,83	75,2	75	13,34	177743 (85,93%)	75,12 - 75,28
SAP (mmHg)	115,8	114	24,08	11049 (93,13%)	115,2-116,39	127,28	125	20,94	177579 (85,85%)	127,15 - 127,41
Temperature (ºC)	37,15	36,9	1,23	10989 (92,62%)	37,1-37,18	36,3	36,2	0,64	165872 (80,19%)	36,3 - 36,31
Stools (nº)	1,01	1	0,9	1286 (10,84%)	0,95 - 1,08	0,89	1	0,94	8000 (3,87%)	0,86 - 0,92
VAS (nº)	1,14	0	2,29	2645 (22,29%)	1,03 - 1,26	1,26	0	2,16	23877 (11,54%)	1,23 - 1,3
Biochemical Variables										
Metabolic										
Albumin (g/dL)	2,99	3	0,63	2290 (19,3%)	2,95 - 3,02	3,56	3,56	0,6	9049 (4,37%)	3,55 - 3,58
Bicarbonate (mEq/L)	24,67	24,4	6,47	1269 (10,7%)	24,2 - 25,14	25,75	25,9	5,91	2363 (1,14%)	25,44 - 26,06
Cholesterol (mg/dl)	128,64	122	53,01	1190 (10,03%)	124,6-132,6	171,05	166	52,73	7772 (3,76%)	169,5-172,59
Capillary glucose (mg/dL)	182,59	160	87,19	3199 (26,96%)	178,62-186,5	150,47	130	67,32	19882 (9,61%)	149,2- 151,7
Blood glucose (mg/dL)	150,78	127	83,79	10537 (88,81%)	148,6-152,89	113,91	101	48,45	98372 (47,56%)	113,5- 114,3
LDH (U/L)	318,35	227	687,57	3498 (29,48%)	288,4 - 348,3	214,16	189	166,37	17293 (8,36%)	210,9-217,42
Total Proteins (g/dL)	5,71	5,7	0,99	1800 (15,17%)	5,65 - 5,77	6,4	6,4	0,78	8649 (4,18%)	6,38 - 6,42
Biomarkers										
CK (U/L)	507,53	76	4056,85	2147 (18,1%)	281,99-733,0	189,69	89	1322,54	21046 (10,17%)	166,2-213,17
Lactate (mmol/L)	2,32	1,7	1,94	1077 (9,08%)	2,16 - 2,47	1,77	1,29	1,53	771 (0,37%)	1,63 - 1,91
Procalcitonin (ng/mL)	12,22	1,37	120,85	4737 (39,93%)	7,7 - 16,75	0,72	0,1	3,22	1646 (0,8%)	0,52 - 0,92
C Reactive Protein (mg/L)	157,53	147,6	100,68	10095 (85,09%)	154,95-160,1	32,37	7,5	56,52	54988 (26,58%)	31,74 - 32,99
Troponin I (ng/L)	698,08	23,7	5647,64	761 (6,41%)	170,7-1225,4	741,99	22,5	4919,38	2730 (1,32%)	499,46-984,5
Renal										
Total Calcium (mg/dL)	8,7	8,7	0,79	10818 (91,18%)	8,68 - 8,72	9,36	9,4	0,58	99505 (48,1%)	9,36 - 9,37
Chloride (mEq/L)	102,19	102,2	5,65	10826 (91,25%)	102,0-102,33	104,7	105	3,8	84249 (40,73%)	104,66-104,7
Creatinine (mg/dL)	1,43	1,11	1,11	10886 (91,76%)	1,41 - 1,46	0,93	0,81	0,56	99953 (48,32%)	0,92 - 0,93
Potassium (mEq/L)	4,28	4,22	0,68	10242 (86,33%)	4,26 - 4,3	4,24	4,22	0,46	94447 (45,66%)	4,24 - 4,25

Sodium (mEq/L)	137,47	137,7	5,55	10811 (91,12%)	137,3 - 137,6	139,32	139,6	3,36	99393 (48,05%)	139,3-139,35
Urea (mg/dL)	61,83	49	43,19	10837 (91,34%)	60,76 - 62,9	36,72	32	22,69	99137 (47,93%)	36,53 - 36,9
Digestive Profile										
Amylase (U/L)	100,06	45	315,38	3250 (27,39%)	85,81-114,31	69,65	55	126,78	23840 (11,53%)	67,53 - 71,76
Direct Bilirubin (mg/dL)	2,03	1,04	2,68	1975 (16,65%)	1,87 - 2,18	1,34	0,66	2,27	4989 (2,41%)	1,26 - 1,43
Total Bilirubin (mg/dL)	1,42	0,79	2,31	7175 (60,48%)	1,35 - 1,49	0,81	0,56	1,26	46142 (22,31%)	0,79 - 0,82
Alkaline Phosphatase (U/L)	139,1	92	182,43	6546 (55,18%)	133,29-144,9	91,67	75	79,98	43352 (20,96%)	90,68 - 92,66
GGT (U/L)	134,67	48	255,94	7196 (60,65%)	126,9-142,44	57,3	22	155,72	48106 (23,26%)	55,48 - 59,13
AST (U/L)	71,3	24	370,29	7151 (60,27%)	60,02 - 82,58	31,92	19	77,95	48096 (23,25%)	31,01 - 32,84
ALT (U/L)	57,78	21	199,57	7452 (62,81%)	51,82 - 63,73	33,4	18	106,61	49682 (24,02%)	32,17 - 34,64
Lipase (U/L)	125,1	18	824,3	3208 (27,04%)	87,61-162,59	55,41	23	403,8	24181 (11,69%)	48,72 - 62,09
Hematological Variables										
Blood Count										
Leukocytes (x10^9/L)	14,38	13,6	9,33	10927 (92,1%)	14,15 - 14,61	9,74	9,04	4,22	105469 (50,99%)	9,71 - 9,77
Neutrophils (x10^9/L)	11,66	10,9	7,17	10848 (91,44%)	11,49 - 11,84	6,59	5,79	3,57	105394 (50,95%)	6,56 - 6,62
Neutrophils (%)	80,15	83,5	13,77	10851 (91,46%)	79,81 - 80,49	66,37	66,6	13,01	105397 (50,95%)	66,26 - 66,47
Lymphocytes (x10^9/L)	1,41	1,05	4,91	10848 (91,44%)	1,29 - 1,53	2,1	1,96	1,85	105395 (50,95%)	2,09 - 2,12
Lymphocytes (%)	11,73	8,7	11,09	10852 (91,47%)	11,45 - 12,0	23,71	23	11,16	105410 (50,96%)	23,62 - 23,8
Eosinophils (x10^9/L)	0,09	0,04	0,21	10886 (91,76%)	0,09 - 0,1	0,16	0,11	0,2	105407 (50,96%)	0,16 - 0,16
Eosinophils (%)	0,82	0,32	1,59	10856 (91,5%)	0,78 - 0,86	1,83	1,3	1,99	105398 (50,95%)	1,82- 1,85
Monocytes (x10^9/L)	0,91	0,81	1,23	10852 (91,47%)	0,88 - 0,95	0,7	0,64	0,35	105403 (50,96%)	0,69 - 0,7
Monocytes (%)	7,06	6,45	4,65	10853 (91,48%)	6,95 - 7,18	7,59	7,3	2,85	105411 (50,96%)	7,57 - 7,61
Basophils (x10^9/L)	0,03	0,02	0,05	10882 (91,72%)	0,03 - 0,03	0,04	0,04	0,03	105411 (50,96%)	0,04 - 0,04
Basophils (%)	0,23	0,15	0,31	10864 (91,57%)	0,23 - 0,24	0,5	0,44	0,36	105404 (50,96%)	0,5 - 0,51
Platelets (x10^9/L)	238,26	218	127,43	10865 (91,58%)	235,1-241,41	244,67	236	80,2	105420 (50,96%)	244,04 - 245,3
PDW (%)	16,35	16,3	1,86	10860 (91,54%)	16,31 - 16,4	16,33	16,3	0,98	105418 (50,96%)	16,33 - 16,34
Median Pla Vol (fL)	7,98	7,8	1,41	10856 (91,5%)	7,95 - 8,02	7,68	7,54	1,13	105416 (50,96%)	7,67 - 7,69
Platelecrit (fL)	0,18	0,17	0,09	10880 (91,71%)	0,18 - 0,18	0,19	0,18	0,71	105430 (50,97%)	0,18 - 0,19
Red blood cells (x10^12/L)	4,01	4,03	0,8	10856 (91,5%)	3,99 - 4,03	4,5	4,53	0,64	105419 (50,96%)	4,49 - 4,5
Hemoglobin (g/dL)	11,89	12	2,28	10857 (91,51%)	11,83 - 11,94	13,43	13,6	1,93	105419 (50,96%)	13,42 - 13,45
Hematocrit (%)	36,68	36,9	7,02	10855 (91,5%)	36,51 - 36,85	40,56	41	5,72	105419 (50,96%)	40,52 - 40,61
MCH (pg)	29,84	30	2,87	10862 (91,55%)	29,77 - 29,91	29,97	30,2	2,52	105424 (50,97%)	29,95 - 29,99
CHgbCM	32,43	32,4	1,34	10860 (91,54%)	32,4 - 32,47	33,12	33,1	1,23	105420 (50,96%)	33,11 - 33,13

RDW (%)	14,1	13,5	2,35	10905 (91,92%)	14,04 - 14,16	12,67	12,2	1,73	105445 (50,98%)	12,66 - 12,69
Carboxihemoglobin	1,55	1,6	0,89	1241 (10,46%)	1,49 - 1,62	1,73	1,5	1,27	2308 (1,12%)	1,66 - 1,8
Metahemoglobin (%)	0,87	0,9	0,44	1234 (10,4%)	0,84 - 0,9	0,84	0,9	0,38	2298 (1,11%)	0,82 - 0,86
MCV (fL)	91,97	92,1	7,64	10865 (91,58%)	91,78 - 92,16	90,47	90,8	6,53	105428 (50,97%)	90,41 - 90,52
Coagulation										
PT act (%)	65,99	68	20,72	9473 (79,85%)	65,44 - 66,54	87,12	90	19,78	76107 (36,79%)	86,94 - 87,3
aPTT (Seg)	33	31,5	8,16	8350 (70,38%)	32,77 - 33,23	31,86	31,1	5,94	68729 (33,23%)	31,8 - 31,92
PT (Seg)	16,61	13,8	12,55	9412 (79,33%)	16,28 - 16,95	12,74	11,4	7,3	76092 (36,79%)	12,67 - 12,81
Fibrinogen (mg/dL)	685,14	674	216,3	8053 (67,88%)	678,9-691,35	472,99	444	150,85	64737 (31,3%)	471,47 - 474,52

Table S2: Structured variables associated with episodes of SE/SS (WSE group)

	VARIABLES	EPISODES (%)	SCORES INCLIDING VARIABLE
1	Age #	11864 (100%)	APACHE-II, SAPS-II, SAPS-III
2	Sex #	11864 (100%)	
3	Heart Rate *	11075 (93,35%)	APACHE-II, LODS, SAPS-II, SAPS-III, SIRS
4	DAP *	11060 (93,22%)	SEPSIS.2
5	SAP*	11049 (92,62%)	LODS, SAPS-II, SAPS-III, SEPSIS.2
6	Temperature *	10989 (92,62%)	APACHE-II, q-SOFA, SAPS-II, SAPS-III, SRIS
7	Leukocytes	10927 (92,10%)	APACHE-II, LODS, SAPS-II, SAPS-III, SRIS
8	RDW	10905 (91,92%)	
9	SatO2 *	10893 (91,82%)	
10	Eosinophils	10886 (91,76%)	
11	Creatinine	10886 (91,76%)	APACHE-II, LODS, SAPS-II/III, SEPSIS.2, SOFA, SEPSIS.3
12	Basophils	10882 (91,72%)	
13	Platelecrit	10880 (91,71%)	
14	Platelets	10865 (91,58%)	LODS, SEPSIS., SOFA, SEPSIS.3
15	MCV	10865 (91,58%)	
16	MCH	10862 (91,55%)	
17	HgbCM	10860 (91,54%)	
18	PDW	10860 (91,54%)	
19	Hemoglobin	10857 (91,51%)	
20	Red blood cells	10856 (91,50%)	
21	Hematocrit	10856 (91,50%)	SAPS-III
22	MPV	10856 (91,50%)	
23	Monocytes	10853 (91,47%)	
24	Neutrophils	10848 (91,44%)	
25	Lymphocytes	10848 (91,44%)	
26	Urea	10837 (91,34%)	LODS, SAPS-II
27	Potassium	10242 (86,33%)	APACHE-II, SAPS-II
28	Chloride	10826 (91,25%)	
29	Calcium	10818 (91,18%)	
30	Sodium	10811 (91,12%)	APACHE-II, SAPS-II
31	Blood glucose	10537 (88,815)	SEPSIS.2
32	CRP	10095 (85,09%)	SEPSIS.2
33	PTact %	9473 (79,85%)	LODS, SEPSIS.2
34	Prothrombin time	9412 (79,33%)	

**3 5**	**aPTT**	**8350 (70,38%**	
**3 6**	**Fibrinogen**	**8053 (67,88%)**	
**3 7**	**ALT**	**7452 (62,81%)**	
**3 8**	**Respiratory Rate***	**7336 (61,83%)**	APACHE-II, SRIS, Q-SOFA
**3 9**	**GGT**	**7196 (60,65%)**	
**4 0**	**Total Bilirubin**	**7175 (60,48%)**	LODS, SAPS-II, SAPS-III, SEPSIS.2, SOFA, SEPSIS.3
**4 1**	**AST**	**7151 (60,27%)**	
**4 2**	**Alkaline phostatase**	**6546 (55,18%)**	

In Table S2, we describe the 42 variables identified in over 55% of the episodes within the WSE group. Notably, the least frequently measured clinical variable is respiratory rate, as reported in many studies. This table illustrates the variables included in the main scores used for sepsis. several of these variables are not incorporated into any scoring system.

Table S3: Relationship between included patients and the presence or absence of SE/SS according to whether they presented in the ED or the rest of the hospital.

PATIENTS	ED (%)*	Others Areas (%)	TOTAL (%)
-WSE	-5.032 (54,10)	-4269 (45,90)	9.301 (100)
-NSP	-169.965 (87,40)	-24.489 (13,60)	194.454 (100)
**TOTAL**	***-174.997 (85,89)***	***-28.758 (14,11)***	***203.755 (100)***

**: $p<0,01$, compared ED with the rest of hospital*

Figure S1.

Origin of patients according to alert systems and electronic medical records.

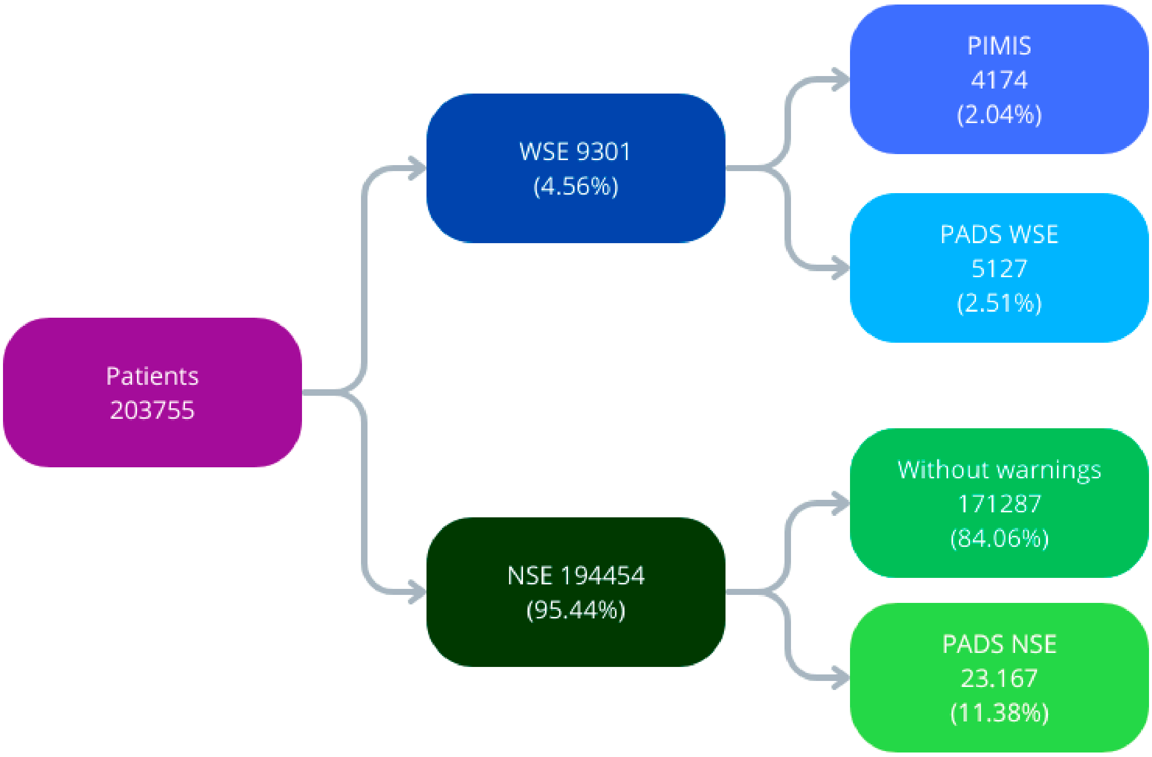

Total patients: 203.755.

With Sepsis patients (WSE) (9.301) were originated from Sepsis Integral Management Informatic Program (PIMIS) and Fixed Rules automatic detection system (PADS). Non septic patients (NSE) originated from negative cases in the automatic fixed rules detection system and validations of the rest of patients that were not detected in both PIMIS or PADS systems.

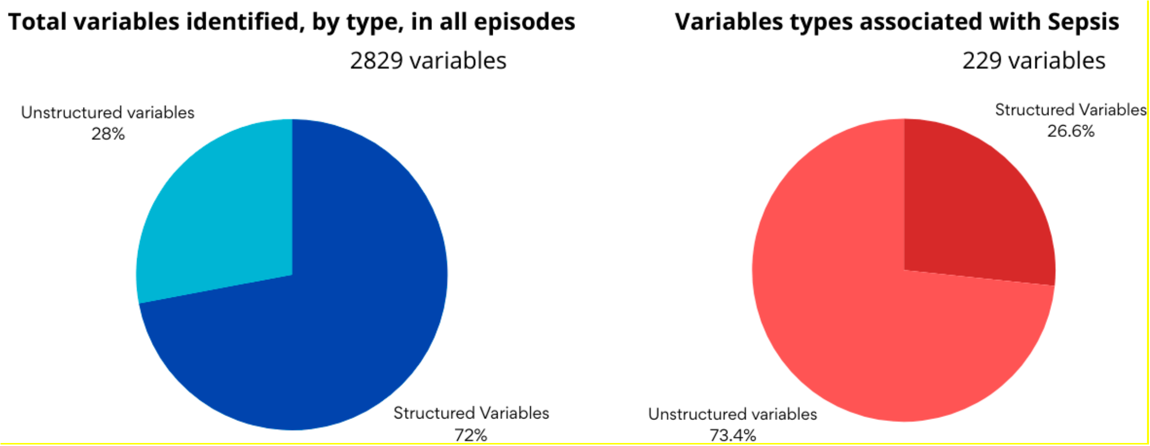

Figure S2. **Types of variables included: structured or unstructured (NLP)**

Table. S4-values of the variables:

The Mann–Whitney–Wilcoxon (MWW) test was applied with a significance threshold of $p < 0.01$, and p-values were subsequently adjusted using the Holm correction to control the family-wise error rate and reduce the likelihood of spurious findings. Variables with $p \geq 0.01$ were not automatically excluded from subsequent machine-learning analyses.

variable	p_value	p_value_adj
**Age**	0.0	0.0
**Platelets**	0.0	0.0
**AST (GOT)**	0.0	0.0
**ALT (GPT)**	0.0	0.0
**Lipase**	7,44E-05	3,72E-03
**White Blood Cells**	0.0	0.0
**Neutrophils**	1,56E-198	2,18E-197
**Neutrophils (%)**	3,50E-90	3,50E-89
**Lymphocytes**	0.0	0.0
**Lymphocytes (%)**	0.0	0.0
**Eosinophils**	0.0	0.0
**Eosinophils (%)**	0.0	0.0
**Monocytes**	0.0	0.0
**Monocytes (%)**	0.0	0.0
**Basophils**	0.0	0.0
**Basophils (%)**	0.0	0.0
**Platelet Distribution Width**	0.0	0.0
**Heart Rate**	0.0	0.0
**Mean Platelet Volume**	0.0	0.0
**Plateletcrit**	0.0	0.0
**Red Blood Cells**	0.0	0.0
**Hemoglobin**	0.0	0.0
**Hematocrit**	0.0	0.0
**Mean Corpuscular Hemoglobin**	0.0	0.0
**Mean Corpuscular Hemoglobin Concentration**	0.0	0.0
**Red Cell Distribution Width**	3,59E-255	6,83E-243
**Carboxyhemoglobin**	3,53E-287	7,41E-285
**Methemoglobin**	9,42E-290	2,07E-276
**Mean Corpuscular Volume**	0.0	0.0
**Prothrombin Activity**	0.0	0.0
**Activated Partial Thromboplastin Time**	0.0	0.0
**Prothrombin Time**	0.0	0.0
**GGT**	0.0	0.0

**Alkaline Phosphatase**	0.0	0.0
**Total Bilirubin**	0.0	0.0
**Direct Bilirubin**	3,83E-110	4,22E-108
**Respiratory Rate**	9,25E-05	3,72E-03
**Glasgow Coma Scale**	6,82E-237	1,09E-234
**FiO2**	4,54E-10	3,18E-08
**Oxygen Saturation**	0.0	0.0
**Diastolic Blood Pressure**	0.0	0.0
**Systolic Blood Pressure**	0.0	0.0
**Temperature**	3,14E-236	4,71E-223
**Bowel Movements**	0.0	0.0
**Pain Scale**	7,56E-59	6,05E-58
**Albumin**	5,15E-61	4,64E-59
**Bicarbonate**	3,91E-278	7,83E-277
**Total Cholesterol**	0.0	0.0
**Capillary Glucose**	2,16E-06	1,29E-04
**Blood Glucose**	0.0	0.0
**Total Proteins**	9,31E-146	1,21E-143
**Creatine Kinase**	1,68E-131	2,02E-141
**Lactate**	0.0	0.0
**Procalcitonin**	5,88E-240	1,00E-226
**C-Reactive Protein**	0.0	0.0
**Troponin I**	0.0	0.0
**Total Calcium**	0.0	0.0
**Chloride**	0.0	0.0
**Serum Creatinine**	0.0	0.0
**Potassium**	0.0	0.0
**Sodium**	0.0	0.0
**Blood Urea**	0.0	0.0
**Serum Amylase**	1,11E-01	3,34E-01
**Fibrinogen**	2,63E-253	4,73E-251
**Blood LDH**	0.724043193	0.724043193
**Mean Arterial Pressure**	0.200885657	0.401771314
